# Study protocol: a randomised placebo-controlled clinical trial to *s*t*u*dy the effect of vitami*n* D suppleme*n*tation on gl*y*caemic control in type 2 Diabetes Mellitus *SUNNY trial*

**DOI:** 10.1186/1472-6823-14-59

**Published:** 2014-07-17

**Authors:** Yvonne HM Krul-Poel, Hans van Wijland, Frank Stam, Edwin ten Boekel, Paul Lips, Suat Simsek

**Affiliations:** 1 From the Department of Internal Medicine, Medical Centre Alkmaar, PO Box 7057, 1815 JD, Wilhelminalaan 12, Alkmaar, The Netherlands; 2 Department of Family Medicine, Alkmaar, The Netherlands; 3 Department of Clinical Chemistry, Haematology & Immunology, Medical Centre Alkmaar, Alkmaar, The Netherlands; 4 Department of Internal Medicine/Endocrinology, VU University Medical Centre, Amsterdam, The Netherlands

**Keywords:** Vitamin D, Type 2 diabetes mellitus, Quality of life, Advanced glycation end products, Insulin resistance

## Abstract

**Background:**

Besides the classical role of vitamin D on calcium and bone homeostasis, vitamin D deficiency has recently been identified as a contributing factor in the onset of insulin resistance in type 2 diabetes mellitus. However, it is uncertain whether vitamin D deficiency and poor glycaemic control are causally interrelated or that they constitute two independent features of type 2 diabetes mellitus. There are limited clinical trials carried out which measured the effect of vitamin D supplementation on glycaemic control.

The objective of this study is to investigate the effect of vitamin D supplementation on glycaemic control and quality of life in patients with type 2 diabetes mellitus.

**Methods/design:**

In a randomised double-blind placebo-controlled trial conducted in five general practices in the Netherlands three hundred patients with type 2 diabetes mellitus treated with lifestyle advises or metformin or sulphonylurea-derivatives are randomised to receive either placebo or 50,000 IU Vitamin D_3_ at monthly intervals. The primary outcome measure is the change in glycated haemoglobin level between baseline and six months. Secondary outcome measures include blood pressure, anthropometric parameters, lipid profile, insulin resistance, quality of life, advanced glycation end products and safety profiles. Quality of life will be measured by The Short Form (SF-36) Health Survey questionnaire. Advanced glycation end products are measured by an AGE-reader.

**Discussion:**

This trial will be the first study exploring the effect of vitamin D supplementation on both glycaemic control and quality of life in patients with type 2 diabetes mellitus. Our findings will contribute to the knowledge of the relationship between vitamin D status and insulin resistance in patients with type 2 diabetes mellitus.

**Trial registration:**

The Netherlands trial register: NTR3154

## Background

Type 2 diabetes mellitus (T2DM), characterized by peripheral insulin resistance and pancreatic beta-cell dysfunction, represents a worldwide epidemic with significant co-morbidity and mortality due to microvascular and macrovascular complications [1]. Although therapies for T2DM and its co-morbidity have improved over the last few decades, the need for new insights for the prevention and management of T2DM remains needed due to the increased impact of the disease. There is accumulating evidence suggesting that vitamin D status plays a role in many non-skeletal functions including diabetes mellitus [2,3]. The prevalence of vitamin D deficiency is increasing with an estimating number of one billion people worldwide [3]. A recent study in the Netherlands among older people revealed a prevalence of vitamin D deficiency of 47.8% (defined as 25 (OH) D < 50 nmol/l) [4].

Vitamin D is a secosteroid that is obtained from dietary sources, either food or supplements, and exposure to sunlight. It needs to be hydroxylated twice to become biologically active. Vitamin D is transported to the liver where it is first hydroxylated by 25-hydroxylase into 25-hydroxyvitamin D (25 (OH) D). This is the major circulating form used as an indicator of vitamin D status. The second hydroxylation occurs in the kidney by 1α-hydroxylase (1α-OHase), a product of the CYP27B1. Here the largest amount of the biologically active form of vitamin D: 1,25 dihydroxyvitamin D (1,25 (OH) 2D) is formed. Besides its classical role in calcium and bone homeostasis, vitamin D deficiency has recently been identified as contributing factor in the onset of insulin resistance in T2DM [5,6]. In a meta-analysis of observational studies a relatively consistent association between low vitamin D status and the prevalence of T2DM or metabolic syndrome was reported [6]. However, due to confounding and selection bias in epidemiological studies, a causal link cannot be established. To determine whether the relation between vitamin D deficiency and glycaemic control is causal in nature, randomised controlled trials with vitamin D supplementation are needed. To date, only few clinical trials examining this relation, with glycaemic control as primary outcome in T2DM, have been performed [7-13]. The results of these clinical trials are inconsistent mostly due to the small sample size, low dose of vitamin D supplementation and short duration of the trials. Recently, a meta-analysis done by George et al. [14] demonstrated a small effect of vitamin D supplementation on fasting glucose and insulin resistance, with no effect on HbA_1c_. However most of these reviewed studies did not include diabetic patients nor had insulin resistance as primary outcome [15-22]. Therefore, adequately powered, randomised placebo-controlled clinical trials with vitamin D supplementation are needed.

The potential mechanisms by which vitamin D can affect glucose metabolism could be the result of a rapid non-genomic effect or slower genomic effect of serum 25 (OH) D: 1) stimulation of insulin release by the increased expression of vitamin D receptor (VDR) as well as the enzyme 1α-OHase in the pancreatic beta-cells; 2) by binding of the 1,25 (OH) 2D - VDR complex to the vitamin D response element of the insulin receptor at tissue level enhancing insulin responsiveness for glucose transport; 3) suppression of the release of pro-inflammatory cytokines that are believed to mediate insulin resistance [2,5,23]. The latter hypothesis is supported by studies showing an association between low serum 25 (OH) D and increased C-reactive protein levels [24]. Indirectly, vitamin D may influence the extracellular and intracellular calcium regulation which is essential in mediating glucose transport in target tissues.

Another hypothesis may be the influence of serum vitamin D on oxidative stress and thereby reducing the formation of advanced glycaemic end products (AGEs). AGEs are a heterogeneous group of compounds formed nonenzymatically by glycation and oxidation of proteins. In patients with T2DM AGE formation is enhanced as a consequence of a hyperglycaemic and free radical rich environment [25]. The AGE accumulation in the skin, as measured by skin autofluorescence, has found to be an independent strong predictor of microvascular and macrovascular complications in both type 1 and type 2 diabetes [26-28]. Until now, solely a study conducted in diabetic rats is available which demonstrated a relation between AGEs and vitamin D [29].

We proposed to start a RCT with the following objectives:

i. To investigate the effect of vitamin D supplementation on glycaemic control and insulin resistance in patients with T2DM.

ii. To investigate the effect of vitamin D supplementation on health related quality of life measured by SF-36 in patients with T2DM.

iii. To investigate the association between skin autofluorescence and serum vitamin D

## Methods/design

### Study design

We designed a double-blind, randomised placebo-controlled clinical trial among patients with T2DM. We randomly assigned three hundred patients into 1:1 ratio to receive a monthly dose of cholecalciferol 50,000 IU or placebo. The follow-up duration is six months. The study will be conducted in five general practices in and around Alkmaar, a city in north-western Netherlands at latitude 52°.

### Participants

All patients with T2DM at the general practices fulfilling the inclusion criteria at their last visit by the general practitioner will be invited for screening for their eligibility.

### Inclusion/exclusion criteria

Adults (≥18 years) with T2DM, diagnosed according to the World Health Organisation, who are treated with lifestyle advises, metformin, or sulfonylurea-derivatives (SU-derivatives), whether or not in combination, were invited for participation. Serum glycated hemoglobin (HbA_1c_) had to be stable and below or equal to 64 mmol/mol (8.0%) for the last three months without recent changes in hypoglycaemic agents.

The exclusion criteria are: an impaired renal function (estimated creatinine clearance < 30 ml/min measured by the MDRD formula: 186 × (serum creatinine)^ -1.154 × (age)^ -0.203 × 0.742 (if the subject is female) or × 1.212 (if the subject is black)), any granuloma forming disorder, known history of renal stones or hypercalcaemia (serum calcium > 2.65 mmol/l), hypo- or hyperphosphatemia, serum 25 (OH) D < 15 nmol/l or > 150 nmol/l, known intolerance for cholecalciferol, using other antidiabetic treatment as mentioned above, insufficient knowledge of the Dutch language, mental retardation or psychiatric treatment for schizophrenia or bipolar disorder, participation in any other trial, being pregnant or lactating, or no signed informed consent.

### Outcomes

The primary outcome of the study is HbA_1c_. Secondary outcomes are insulin resistance and beta-cell function measured by the homeostasis model of assessments (HOMA) and quantitative insulin sensitivity check index (QUICKI). Further secondary outcomes include the alteration of serum 25 (OH) D over time, blood pressure, lipid profile, parathyroid hormone, calcium, thyroid function, AGE accumulation in the skin, safety profile of vitamin D supplementation, urine analysis for microalbuminuria, and quality of life assessed by The Short Form (SF-36) Health Survey questionnaire.

### Sample size estimation

It was calculated that 126 patients with T2DM would be required in this trial to demonstrate a significant difference at 80% power and 5% significance. Power calculations were based on the literature and aimed at a difference of 0.5% in HbA_1c_ value in the treated group as compared to the placebo group with a standard deviation of 1.0% [30]. With an expected rate of 50% vitamin D deficient participants, 252 subjects would be required. According to an expected drop out rate of 20%, 300 subjects will be recruited.

### Randomisation

A double-blind randomised placebo-controlled trial design has been chosen. Randomisation for the parallel treatment phase will be carried out by the pharmaceutical department after checking the inclusion- and exclusion criteria. The patient will be assigned to the treatment order as defined by the code. The patients will be randomised 1:1 according to the method of block randomisation with a block size of 10. No stratification is used. The study medication will be delivered in similar flagons and labels with its own sequence number. The allocation sequence number will be kept by the pharmacist in a secure place during the course of the study.

### Intervention

In this trial the dose of vitamin D (cholecalciferol) being supplemented is 50,000 IU. The consumption frequency will be once a month (equivalent to ~1667 IU/day in the treatment group) for six consecutive months. We chose the oral route of administration. Cholecalciferol is supplied in a vial of 8 ml containing 50,000 IU per ml, meaning the patients will use 1 ml per month. The placebo will be identical in taste, texture and appearance as the active supplement. The vitamin D supplement and placebo are manufactured and packed by the pharmacy of the Meander Medical Centre, Amersfoort, the Netherlands. Both the active supplements and the placebo will be given to participants in a translucent flagon with only their sequence number on the label. Labeling of the flagons will be done by the same person doing the randomisation and allocation sequence. A pipette of 1 ml will be delivered by the vial to make sure all patients use the right dose of the study medication. During their first visit patients will be instructed how to take in the study medication correctly with the pipette. To ensure compliance during the study, reminders about the intervention appointments will be sent regularly via email of phone calls.

The safety of vitamin D supplementation has well been established. The recommended dose for adults is 600–800 IU/day, depending on age, with an upper limit of 4,000 IU/day [31]. Toxicity of vitamin D supplementation did not occur with a dose of 10,000 IU/day [32]. Side effects (hypercalcaemia, hypercalcuria, renal stones, gastro-intestinal symptoms or hypersensitivity symptoms) of vitamin D supplementation are rare. For the safety of all patients serum creatinine and calcium are measured at three months. Patients with known hypercalcaemia are excluded for this reason. A 24 hour telephone number is available in the case hypersensitivity symptoms occur.

### Handling and storage of data and documents

For each participant a Case Record Form (CRF) is provided by the sponsor of the trial. All personal data are stored on a coded drive which is only available to the study coordination team. These documents will be stored in the hospital for a minimum of ten years. The Subject Identification Codes must be kept for at least 15 years. All these requirements are in accordance with the ‘Wet Medisch Onderzoek met Mensen’.

### Statistical analysis

The primary efficacy analysis to explore the intervention effect on glycaemic control will be based on the intention-to-treat (ITT) method, in which all randomised patients for whom outcome data are available, will be recorded. In the ITT analysis the patients will be analysed according to their original group assignment, whether or not they accepted and/or adhered to the intervention. This analysis avoids the possibility of any bias associated with loss, misallocation or non-adherence of participants. If there will be a substantial difference between those allocated to receive an intervention and those who actually received it (and adequately adhere to it), we will perform an additional analysis adjusting for actual treatment received (‘per protocol’ analysis). The results of the ‘per protocol’ analysis will be compared with the ITT analysis and the numbers involved will be precisely described.

All raw data will be entered into SPSS software (version 20.0, SPSS Inc, Chicago, IL). Ten percent of all data will be double-entered to check for duplication and outliers before starting statistical analyses.

Baseline characteristics of the study patients will be summarised separately for each randomised group. Dichotomous and categorical data will be presented as number and percentages of patients within both groups. Continuous variables will be checked for normality and will be presented in terms of means and standard deviation if normally distributed, or when a variable has a skewed distribution as median, 25th and 75th percentiles. Nominal variables including subgroups will be analysed using the Chi-squared test, ordinal variables by the Mann-Witney test or Kruskall-Wallis test and continuous variables by the *t*-test or analysis of variance model (ANOVA).

The primary efficacy endpoint, HbA_1c,_ will be compared between placebo and treatment group using a repeated measures analysis of variance with adjustment for baseline characteristics. For secondary outcomes a paired *t*-test, Wilcoxon’s test or repeated measures analysis of variance will be performed. Delta values will be calculated to compare the differences in means over time between the treatment and placebo group.

Correlation coefficients will be measured using Pearson or Spearman, depending on the level of measurement. A logistic and/or linear regression model will be used for multivariate analysis to relate an outcome variable to causal variables. The multivariate analysis will be used to rule out the possible role of confounders for vitamin D deficiency as season of measurement, maternal age, ethnicity, diet, physical activity and BMI.

Subgroup analyses will be performed by stratifying vitamin D into three groups: deficient (15–49 nmol/l), insufficient (50–74 nmol/l), and sufficient (75–150 nmol/l). Furthermore, subgroup analyses for vitamin D deficient patients (serum 25 (OH) D 15–35 nmol/l), and poor glycaemic control (HbA_1c_ ≥ 53 mmol/mol) will be performed using the same method as described for the primary analysis. A p-value < 0.05 will be considered as statistical significant.

### Funding/ethics

This trial was approved by the Medical Ethics Committee of North-Holland, the Netherlands. No funding is given for the trial.

## Results

### Study procedure

The study will be divided into two phases: Phase I and Phase II.

#### Phase I

Patients with T2DM who met the inclusion criteria at their last visit at the general practice will be invited through a brief introduction letter for participation. All patients will be approached by phone in three weeks after receiving the letter whether or not they are interested for participation. If interested, the extensive patient information form will be given and an appointment will be scheduled at the general practice for informed consent and start of the trial.

#### Phase II

**Figure 1:**
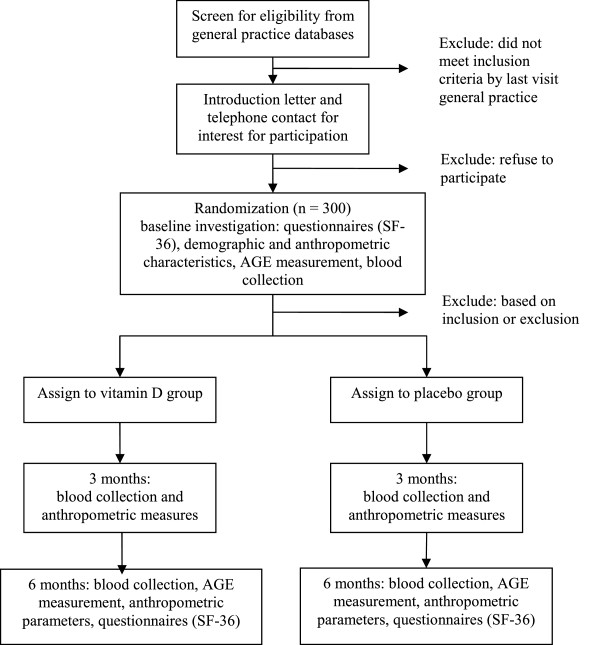
Recruitment flow chart.

The trial has a total of three visits at the patient’s own general practice (Figure 1). The initial visit includes informed consent, assessment of eligibility, questionnaires regarding demographics, sunlight exposure, physical activity, dietary intake (fish and dairy products), diabetes history and medication, co-morbidity and health related quality of life (SF-36). Anthropometric parameters and skin AGE accumulation will be measured. Blood collection will be collect at this stage. The study medication will be given for six months with a prior instruction. The second visit at three months is for safety assessment and observation of consumption. This includes: discussion of any adverse events, any change in medication, control of the ingestion of the study medication, blood collection (including serum calcium, renal function, serum 25 (OH) D and HbA_1c_). The final visit is at the end of six months, when questionnaires and anthropometric measurements are repeated and the last set of blood samples are collected (Table 1). The inclusion of all patients will be spread over one year to prevent large seasonal influences, as well as the results will be adjusted for seasonal influences.

**Table 1:** Outcome variables and time of measurement (n = 300)

**Variables**	**Months**		
	0	3	6
Informed Consent	x		
Sociodemographics	x		
Medical and family history	x		
Diet, sun exposure, physical activity	x		x
Advanced Glycation End products (AGEs)	x		x
Questionnaire SF-36	x		x
Medication alterations		x	x
Adverse events		x	x
***Anthropometry***			
Height, weight, BMI	x	x	x
Blood pressure	x	x	x
Waist/hip circumference	x		x
***Blood analysis***			
Serum 25 (OH) D	x	x	x
HbA_1c_	x	x	x
Fasting blood glucose	x	x	x
Fasting blood insulin	x	x	x
C-peptide	x	x	x
Serum creatinine	x	x	x
Lipid profile	x		x
Serum calcium	x		x
Serum phosphate	x		x
Serum albumin	x	x	x
Serum PTH	x		x
Serum TSH	x		x
AST	x		x
ALT	x		x
Alkaline phosphatase	x		x
γ-GT	x		x
Hb, Platelets, Leukocytes	x		x
C-reactive protein	x		x
Urine analysis	x	x	x

All participants will be informed of their anthropometry and blood pressure measurement at the start and during the course of the trial. The blood test results during the course of the trial will only be discussed when these are abnormal and lead to premature termination of the trial. The following criteria are drawn for premature termination of the trial: hypercalcaemia (calcium > 2.65 mmol/l), onset of any granuloma forming disease, serum 25 (OH) D < 15 nmol/l or > 250 nmol/l, impaired renal function (eGFR < 30 ml/min), hypersensitivity for cholecalciferol, onset of urolithiasis, change in hypoglycaemic agents or HbA_1c_ > 69 mmol/mol (8.5%). After completion of the trial, all participants will be informed of their blood results. In case of vitamin D deficiency (serum 25 (OH) D < 50 nmol/l) at the end of the trial, supplementation with cholecalciferol will be started. The patients will be informed by letter in which group they participated after every patient completed the trial.

### Anthropometric parameters, AGE measurement and health related questionnaire

Height and weight will be measured using a stadiometer (up to 2 meters) and a weighing scale (200 kilograms). Weight will be measured without shoes and with light clothes in the morning. Blood pressure will be measured using a sphygmomanometer. The blood pressure will be measured at the right arm whilst the patient is sitting for at least 5 minutes. Tape-measurements will be performed for waist and hip circumferences. Body Mass Index (BMI) will be calculated by dividing weight through the square of height. Waist to hip ratio will be calculated by the formula: waist circumference/hip circumference. The AGE accumulation in the skin will be measured using an AGE reader (Diagnoptics Technologies B.V., Groningen, The Netherlands). This is a non-invasively device that can assess the tissue accumulation of AGEs using fluorescence of ultraviolet light in human tissue. The forearm is put on the machine and the concentration is assessed by ultraviolet light. This procedure takes 3 minutes and will be done at baseline and at the final visit.

SF-36 health survey will be used to assess health-related quality of life on emotional and physical wellbeing. The patients will receive the questionnaire at baseline and at the end of the trial with a self-addressed envelope.

### Blood collection

Venous blood sampling will be drawn at 0, 3 and 6 months by registered staff nurses using a sterile vacutainer needle between 8.00 and 9.30 am. All participants will be asked to fast overnight for at least 8 hours before blood collection. Blood serum will be used for the analysis of HbA_1c_, C-peptide, fasting insulin, calcium, phosphate, albumin, PTH, liver enzymes, lipid profile, C-reactive protein, TSH, platelets count, leukocytes and hemoglobin and 25 (OH) D ((iSYS automated immunoanalyser (IDS GmbH, Frankfurt, Germany)). The total 25-OH vitamin D assay detects 25-OH VD3 and 25-OH VD2, both with a specificity of 100%. The quality of the test is controlled by applying Westgard QC-rules on 3 different QC-samples [33]. The accepted interassay coefficients of variation are <10% for all 3 QC-samples (low, medium and high level). TSH and intact-PTH will be determined on a Beckman Coulter UniCel DxI 600 Synchron Access immunoanalyser (Beckman Coulter Nederland B.V., Mijdrecht, the Netherlands). Calcium, phosphate, glucose, albumin, liver enzymes, C-reactive protein and lipid profiles will be determined on a Beckman Coulter UniCel DxC 860i Synchron Clinical system chemistry analyzer. C-peptide and insulin will be determined on Siemens Immulite XPi automated immunoanalyser (Siemens Medical Solutions Diagnostics B.V., Breda, the Netherlands). HbA_1c_ will be measured on a HA-8180 automated HPLC system Menarini (Florence, Italy). The total white blood cell count, platelets count and haemoglobin content will be performed with an automated Sysmex XE-2100 blood cell counter (Sysmex Coorperation, Kobe, Japan).

Fasting blood glucose will be measured in blood plasma. Homeostasis model assessment of insulin resistance (HOMA-IR), calculated by fasting glucose (mmol/l) x fasting insulin (IU/ml)/22,5 and QUICKI, calculated by 1/log (fasting insulin (IU/ml)) + log (fasting glucose (mg/dl)), will be used to evaluate insulin resistance. Pancreatic beta-cell function will be assessed through the HOMA-B, calculated by (20 x fasting insulin (IU/ml))/(fasting glucose (mmol/l) – 3,5). All assays will be performed according to the manufacturer’s instructions and carried out by the central chemical laboratory of the Medical Centre Alkmaar, the Netherlands. This laboratory is a certified CCK laboratory. Aliquots of serum and plasma will be stored at -70/80’C for future research questions.

### Adverse events

All serious adverse events (SAEs) will be reported through the web portal *ToetsingOnline* to the accredited Medical Ethics Committee that approved the protocol, within 15 days after the sponsor has first knowledge of the serious adverse reaction. SAEs that result in death or are life threatening episodes should be reported expedited. The expedited reporting will occur not later than 7 days after the responsible investigator has first knowledge of the adverse reaction. This is for a preliminary report with another 8 days for completion of the report.

## Discussion

The main purpose of this study is to measure the effect of vitamin D supplementation on glycaemic control and health related quality of life in patients with T2DM. There is widespread interest in the potential causal role of vitamin D on the pathogenesis and progression of T2DM. We hope this study will give new insight into this causality. Until now conflicting results are seen in observational studies and the few clinical trials performed, which could not confirm a causal association [7-14]. Factors for the lack of effect found in these studies includes: short trial duration, relatively low doses of vitamin D supplementation whether or not combined with calcium supplements, and heterogeneous study populations.

This trial has several strengths: the study sample size allows us to perform a subgroup analysis of vitamin D deficient patients. Taken into account that only in vitamin D deficient patients an effect of vitamin D supplementation on glycaemic control will be found, we chose to recruit double the number of participants as calculated with the power estimation, hypothesizing that 50% of the included patients will be vitamin D deficient (serum 25 (OH) D < 50 nmol/l). Secondary, the relatively high dose chosen for intervention and the duration of the trial are strengths of our study. The dose of vitamin D supplementation of 50,000 IU of vitamin D per month (daily equivalent ~1667 IU) is based on the recommendations of the Institute of Medicine and on the study done by van Groningen et al. [31,34]. In this latter study, performed in the Netherlands, it was demonstrated that a cumulative dose of 100,000 IU given in 2 months increases the serum 25 (OH) D meanly with 29 nmol/l. Assuming that the mean serum 25 (OH) D in our study population will be around 50 nmol/l at baseline, the serum 25 (OH) D should be raising to a sufficient status (≥75 nmol/l) in two months. To measure a difference in HbA_1c_ level we hypothesize that the maximal effect will be seen at least at six months, regarding the fact that the red blood cells circulate about 100 days in the blood and HbA_1c_ levels takes around six weeks to change. Furthermore, this is the first study examining the association between vitamin D status and skin autofluorescence value as well as the effect on skin AGEs after vitamin D supplementation. For this secondary outcome the duration of our trial will be a limitation, regarding the stability and long half-life of skin AGEs with could be the cause to find no effect of vitamin D supplementation on skin AGEs.

The results of this trial should provide more insight into the potential causal association between vitamin D status and glycaemic control in patients with T2DM.

### Trial status

This study is in the process of participant recruitment.

## Abbreviations

1,25(OH)2D: 1,25-dihydroxyvitamin D; 25(OH)D: 25-hydroxyvitamin D; 1α-OHase: 1-α-hydroxylase; AGE: Advanced glycation end products; ANOVA: Analysis of variance; BMI: Body mass index; HbA_1c_: The standard measure glycaemic control, measuring glycaemic control over the last six to eight weeks; HOMA: Homeostatic model assessment; IU: International units; PTH: Parathyroid hormone; QUICKI: Quantitative insulin sensitivity check index; SF-36: Short form 36 of health survey; VDR: Vitamin D receptor.

## Competing interests

The authors declare that they have no competing interests.

## Authors’ contributions

SS designed the initial idea of this work, which was further developed by the other authors. YK and SS coordinated the study. YK and SS advised on statistical analysis in cooperation with the biostatistician mentioned in the acknowledgements. YK and CS coordinated recruitment, participant management and data-collection. YK drafted the manuscript. All authors reviewed the study protocol and approved the final manuscript.

## Pre-publication history

The pre-publication history for this paper can be accessed here:

http://www.biomedcentral.com/1472-6823/14/59/prepub
